# Teaching under pressure: assessing burnout among high school teachers

**DOI:** 10.11604/pamj.2024.48.78.42914

**Published:** 2024-06-28

**Authors:** Hicham Guider, Fatine Hadrya, Mohammed Amine Lafraxo, Youssef El Madhi, Abdelmajid Soulaymani, Abdelrhani Mokhtari, Hinde Hami

**Affiliations:** 1 Laboratory of Biology and Health, Faculty of Science, Ibn Tofail University, Kenitra, Morocco,; 2 University Hassan First of Settat, Higher Institute of Health Sciences, Health Sciences and Technologies Laboratory, Settat, Morocco,; 3 Higher Institute of Nursing Professions and Health Techniques, Oujda, Morocco,; 4 Regional Center for Education and Training Professions, Rabat, Morocco

**Keywords:** Academic stress, burnout, determinants, teacher well-being, challenges

## Abstract

**Introduction:**

the increasing demands and responsibilities placed upon teachers are causing overwhelming stress. As a result, many teachers struggle with managing their workload effectively, which increases their susceptibility to burnout. This study aims to investigate burnout among high school teachers in Tetouan, Morocco, and explore the various contributing factors.

**Methods:**

a cross-sectional study was conducted involving teachers who completed the Maslach Burnout Inventory and a comprehensive questionnaire covering personal attributes and professional responsibilities. We then used statistical software to analyze the collected data. **Results:** the study, involving 258 high school teachers, highlights a significant prevalence of burnout, with levels ranging from moderate to high. Particularly notable is the prevalence of emotional exhaustion, which affected 43% of the participants, whereas depersonalization (46%) and personal accomplishment (47%) exhibited relatively lower levels of concern. In addition, a thorough analysis revealed significant correlations between burnout rates and contributing factors, including gender, number of classes taught, class size, weekly working hours, and engagement in regular physical activity.

**Conclusion:**

this study highlights the substantial prevalence of burnout among high school teachers, emphasizing the urgency of teacher well-being. It enhances the understanding of the association between burnout and different work-related and lifestyle factors, enabling the identification of appropriate interventions. The interaction between burnout and these factors implies the need to further investigate gender-specific and workload-related preventive measures.

## Introduction

Professional exhaustion syndrome, commonly known as burnout, is a global problem with far-reaching implications for professionals in different sectors [1-4]. This syndrome is characterized by persistent symptoms that lead to negative psychological experiences, distorted motivations, and deterioration of psychophysical well-being [5]. The consequences extend far beyond the individual, profoundly affecting the ability to work and the overall quality of life of those affected. In particular, the field of education is emerging as a particularly vulnerable domain to the challenges posed by burnout [6].

Within the field of contemporary educational psychology, the psychological well-being of teachers is of paramount importance. The teaching profession inherently involves significant levels of informational and emotional stress, making educators particularly vulnerable to burnout [7,8]. Extensive research has been conducted to shed light on the profound effects of burnout on professionals, with teachers identified as a particularly vulnerable group [9]. The consequences of burnout among teachers are multifaceted and have a significant impact on their professional lives. These consequences manifest themselves in reduced performance, diminished passion for their chosen profession, and an overall decrease in effectiveness [10].

This study explores the phenomenon of burnout among high school teachers and assesses its various aspects in association with many factors. Our hypothesis suggests that teachers experience heightened levels of emotional exhaustion and depersonalization, along with a reduced sense of personal accomplishment. In addition, our proposal anticipates a correlation between burnout rates and several influential factors.

## Methods

**Study design:** a cross-sectional study was conducted in March and April 2021 to assess the prevalence of burnout among high school teachers and its association with various contributing factors.

**Study setting:** this study focused on teachers teaching experimental sciences in Tetouan, northwest Morocco, located approximately 60 km southeast of Tangier and adjacent to the Strait of Gibraltar.

**Participants:** Tetouan, with its 15 high schools, provides an ideal setting for our investigation into the teaching of experimental sciences. We identified 467 teachers in this specialty. A simple random sampling method was employed to select 8 high schools, ensuring a diverse and representative subset of this population. This approach was chosen to generate robust and generalizable data, resulting in a target group of 258 experimental science teachers. Of these, 147 completed our questionnaire, yielding a response rate of 57%. Such a rate suggests a high level of engagement among the participants, underscoring the relevance of our study to their professional practice. The timing of the questionnaire distribution was carefully planned to coincide with teachers' schedules, maximizing response rates. To ensure the integrity of our study, rigorous ethical measures were implemented, including obtaining informed consent and guaranteeing anonymity for all participants, thus bolstering the validity and reliability of our findings.

**Data sources and measurements:** this study evaluated teachers´ burnout using the Maslach Burnout Inventory (MBI). The MBI measures emotional exhaustion (EE), depersonalization (DP), and personal accomplishment (PA), which are complex aspects of burnout [11]. Specifically tailored to the field of education, the MBI-Educators Survey was used in this study. This survey evaluated professional burnout using 22 Likert-type items ranging from “never” to “every day” [12]. The assessment yielded a classification of “low,” “moderate,” or “high” for each dimension. Burnout is classified according to the presence of at least two pathological dimensions, with a score indicating high emotional exhaustion, depersonalization, or low personal accomplishment as adequate criteria for classification. Burnout is classified as “moderate” when two dimensions display pathological traits, while it is classified as “high” when all three dimensions present such attributes. This study employed a validated French version of the MBI, as established by Dion and Tessier [13]. It is worth noting that the questionnaires used were thoroughly designed to collect information on these variables. A comprehensive survey was conducted to gather data on multiple factors, including gender, age, marital status, number of children, education level, initial career assignment cycle, specialization, professional experience, number of classes taught, number of students per class, weekly working hours and regular participation in sports.

**Data analysis:** our study on burnout among experimental science teachers in Tetouan, used a comprehensive statistical approach to address our research questions. This study aimed to elucidate the relationship between burnout and various professional and personal factors. Initially, we conducted a frequency analysis to delineate the demographic and occupational characteristics of our sample. Subsequently, the reliability of the measurement instrument was assessed by calculating Cronbach's alpha coefficient, with a threshold value of 0.7 indicating acceptable internal consistency for the survey's scales. To examine the correlations between burnout dimensions and the quantitative characteristics, the Spearman rank correlation coefficient was applied. This choice was informed by the non-normal distribution of the data. The use of Spearman´s rho enabled us to identify statistically significant correlations while acknowledging the non-parametric context and setting a significance level of 5%. This analytical process was crucial for identifying significant associations, providing a strong foundation for in-depth interpretation in the results section.

## Results

**Demographic and professional profile of participating teachers:** upon analysis of Table 1, it is evident that female teachers held a slightly higher prevalence at 52%. A significant portion of teachers fell within the age bracket of 31 to 40 years, which encompassed 39% of the total. Additionally, 78% of teachers were married, with an average of two children per teacher (30%). The majority of teachers began their teaching careers in high schools, accounting for 77% of the respondents. They have significant subject-specific experience in the field, with 26% of them having worked for more than 20 years. A notable proportion of them held position 11 within the Moroccan educational system, making up 36% of the respondents. Academically, 50% of teachers held a bachelor´s degree as their highest educational qualification. The majority of teachers (63%) managed between four and six classes, with an average class size exceeding 30 students. Additionally, teachers usually spend approximately 20-21 hours per week fulfilling their teaching responsibilities (62%).

**Table 1:** demographic and professional profile of participating teachers

Category	N (%)*
**Gender**	
Female	76 (52)
Male	71 (48)
**Age group**	
21-30 years	20 (14)
31-40 years	58 (39)
41-50 years	38 (26)
Over 50 years	31 (21)
**Marital status**	
Married	115 (78)
Unmarried	27 (18)
Divorced	5 (4)
**Number of children**	
None	40 (27)
1 child	24 (16)
2 children	44 (30)
More than 2 children	39 (27)
**Level of education**	
Bachelor’s degree	73 (50)
Master’s degree	49 (33)
Doctorate	9 (6)
Other	16 (11)
**Teaching subject**	
Mathematics	55 (37)
Physics/Chemistry	44 (30)
Life sciences	48 (33)
**Years of teaching experience**	
1-5 years	10 (7)
6-10 years	36 (24)
11-15 years	37 (25)
16-20 years	26 (18)
Over 20 years	38 (26)
**Number of classes taught**	
1-3 classes	21 (14)
4-6 classes	92 (63)
More than 6 classes	34 (23)
**Class size**	
10-20 students	21 (14)
21-30 students	41 (28)
More than 30 students	85 (58)
**Weekly working hours**	
14-16 hours	16 (11)
17-19 hours	40 (27)
20-21 hours	91 (62)
**Regular physical activity**	
Yes, regularly	31 (21)
Rarely	80 (54)
No	36 (25)

* N (%): number of participants (percentage)

**Reliability of the burnout scale:** the assessment of the burnout scale´s reliability provided valuable findings. The emotional exhaustion scale displayed exceptional reliability, with a Cronbach´s alpha of 0.857, confirming the accuracy of its burnout item measurement. The depersonalization scale also demonstrated high reliability, with a Cronbach´s alpha of 0.713. However, the analysis of the depersonalization scale demonstrated a significant compromise in reliability due to the removal of certain items. The inclusion of each item is essential to ensure precise measurement. Notably, the personal accomplishment scale exhibited a Cronbach´s alpha of 0.862, indicating a high level of reliability in its evaluation of burnout (Table 2).

**Table 2:** Maslach burnout inventory scale reliability: alpha coefficients for the burnout components

EE Scale									
Items	1	2	3	6	8	13	14	16	20
Alpha	0.835	0.845	0.835	0.860	0.832	0.841	0.846	0.847	0.840
**DP scale**									
Items	5	10	11	15	22				
Alpha	0.650	0.650	0.600	0.683	0.729				
**PA scale**									
Items	4	7	9	12	17	18	19	21	
Alpha	0.854	0.855	0.844	0.847	0.831	0.847	0.845	0.838	


MBI: Maslach burnout inventory; EE: emotional exhaustion; DP: depersonalization; PA: personal accomplishment

**Figure 1:**
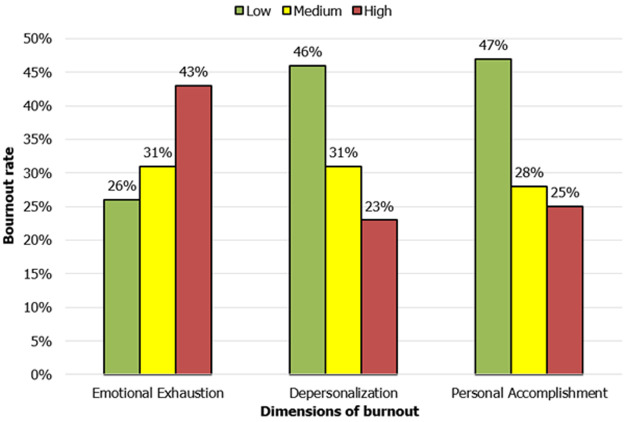
levels of emotional exhaustion, depersonalization, and personal accomplishment experienced by teachers

**Burnout analysis:** regarding emotional exhaustion, 43% of teachers indicated high levels, 31% reported moderate levels, and 26% expressed low levels. For depersonalization, 46% of teachers displayed low levels, 31% exhibited moderate levels, and 23% exhibited high levels. As for personal accomplishment, 47% of participants conveyed low levels, 28% reported moderate levels, and 25% achieved high levels (Figure 1).

**Correlations between burnout dimensions and work-related factors:** the correlation analysis presented in Table 3 displays significant associations. Significant correlations emerge within the emotional exhaustion scale with two factors: gender (0.179) and weekly work hours (0.209), indicating proportional fluctuations with this dimension. Additionally, the emotional exhaustion scale shows an inverse correlation with the variable “regular physical activity” (-0.288), implying opposite trends. The depersonalization dimension revealed a direct correlation with the number of classes taught (0.227). Concerning the personal accomplishment dimension, a positive correlation was obvious with “weekly work hours” (0.173), indicating proportional variations. However, the personal accomplishment dimension demonstrates a negative correlation with the “number of classes taught” (-0.223) and “number of students in the class” (-0.255), indicating inverse trends with these dimensions.

**Table 3:** correlations between burnout dimensions and various factors among teachers

Category	EE scale	DP scale	PA scale
Gender	0.179*	-0.055	0.137
Age	-0.033	-0.133	0.013
Marital status	0.040	0.068	-0.012
Number of children	-0.040	-0.022	0.018
Level of education	-0.081	-0.162	0.124
Teaching subject	0.104	-0.114	0.085
Years of teaching experience	0.098	0.009	-0.066
Number of classes taught	0.144	0.227**	-0.223**
Class size	0.141	0.114	-0.255**
Weekly working hours	0.209*	-0.138	0.173*
Regular physical activity	-0.288**	-0.146	0.092

EE: emotional exhaustion; DP: depersonalization; PA: personal accomplishment; *the correlation is significant at the 0.05 level (2-tailed); **the correlation is significant at the 0.01 level (2-tailed).

## Discussion

Our study revealed a significant prevalence of burnout among teachers in Tetouan, with 39% reporting high levels of burnout. This finding underscores the pressing need for strategies to mitigate workplace stress in the educational sector. In addition, a notable 43% of teachers reported increased emotional exhaustion, and 47% experienced a decrease in personal accomplishment, aligning with the core dimensions of burnout identified in the literature [2,14]. Emotional exhaustion, as a core dimension of stress, and personal accomplishment, which relies on self-assessment, align with a discernible pattern in burnout research [15]. The heightened emotional exhaustion experienced by teachers may stem from discord between their professional values and the perceived undervaluation of their work. Another contributing factor to consider is the presence of an inadequate reward system, as suggested by Piechurska-Kuciel [16]. This context may stem from the significant workload imposed on teachers in conjunction with limited resources and delayed recognition and rewards [17,18]. The consequences of this scenario often result in teacher emotional exhaustion, leading to disengagement from the teaching and learning process and the development of depressive symptoms. These findings support previous research [19,20].

Gender also emerges as a relevant factor linked to burnout. Extensive research indicates that women typically have higher levels of emotional exhaustion than men, a pattern consistent with previous studies [10,21-23]. This gender difference can be attributed to the specific and numerous responsibilities that women usually handle, both in the school setting and at home. Additionally, women tend to exhibit emotional distress through psychosomatic symptoms, in line with cultural norms [24]. Nevertheless, some studies suggest that gender may not be a significant factor in burnout [25], and in some cases, men experience higher levels of emotional exhaustion than women [26].

Age is an additional factor linked to teacher burnout. Studies indicate that teachers over the age of 30 typically experience greater emotional exhaustion than their younger colleagues, which aligns with the results of earlier studies [9,22]. This finding agrees with prior research that postulated that younger teachers are more resilient to burnout [27]. The burnout experienced by teachers may be attributed to challenging teaching conditions when dealing with new generations of students. However, Yektatalab *et al*. [28] identified a significant positive correlation between burnout subscales and younger age, indicating that further investigation is required to understand the relationship between age and burnout. On the other hand, other studies have suggested that young age is a risk factor for workers [21,23].

Several contributing factors underlie the emergence of teacher burnout, such as weekly working hours and class size. According to this study, teachers who worked between 20 and 21 hours per week experienced heightened emotional exhaustion compared with those who worked fewer than 20 hours per week. These findings align with prior research that links extended work hours to an increase in stress and consequent elevation of burnout rates among teachers [22]. This correlation extends beyond the teaching field to other personal workers [29]. Yestiana *et al*. found that both work volume and workload significantly contribute to burnout among nurses in public hospitals [30]. Additionally, class size is another critical factor linked to teacher burnout. Studies indicate that teachers who face large class sizes report more emotional exhaustion than those who teach small groups. This finding is consistent with previous studies that highlight the increased commitment and attention demanded by large groups of students. Moreover, Ibáñez *et al*. observed a positive correlation between heightened workload and burnout, indicating that large class sizes could negatively impact teacher well-being [22].

Teaching is an inherently demanding profession that requires unwavering dedication to both students and work conditions, making teachers susceptible to burnout, as supported by the findings of our study and previous research [31-36]. Although our study recorded lower burnout rates among teachers than among Moroccan healthcare professionals [37-40], the concern remains worrisome and necessitates targeted interventions in the educational sector. The prevalence of burnout among teachers, although comparatively lower, is still significant enough to necessitate the same level of attention and urgency as in other healthcare professions.

Our research presents new, context-specific findings regarding teacher burnout in Tetouan, offering groundwork for developing customized amelioration strategies for this cohort. Furthermore, these findings facilitate the development of specific interventions to address the distinctive challenges experienced by teachers in the region. Nevertheless, the limited scope and particular focus of our study on a specific group of teachers may restrict the generalizability of our conclusions. For a more comprehensive understanding, future research endeavors should adopt a longitudinal approach. This will illuminate the trajectories of burnout and facilitate tracking its progression over time, which are crucial steps for optimizing the effectiveness and timing of interventions.

## Conclusion

Our study underscores the urgent need for actionable measures from policymakers and educational authorities. Robust, evidence-based support systems must be prioritized to address the myriad risk factors that contribute to teacher burnout. The well-being of teachers demands urgent attention and warrants further research to analyze the complex psychological and societal determinants of teacher stress. Future studies must develop nuanced and context-aware strategies for prevention and intervention to ensure their effectiveness in diverse educational environments. The findings not only highlight the significant challenges of teacher burnout but also lay the groundwork for more in-depth explorations aimed at crafting effective solutions for this pervasive issue.

### What is known about this topic

- *Teacher burnout is a worldwide issue that has a negative impact on both psychological health and teaching quality;*
- *In Morocco, the intensity of educational demands necessitates further research on teacher burnout;*
- *A comprehensive understanding of the multiple facets of teacher burnout is essential for developing effective interventions*.

### What this study adds

- *This study delineates actionable correlations between specific modifiable workplace factors, such as workload, and teacher burnout, providing empirical evidence for the design and implementation of targeted educational policy reforms and teacher support initiatives;*
- *It also quantifies the positive impact of lifestyle changes, such as regular physical activity, on mitigating burnout; this substantiates the necessity for integrating wellness programs into teacher development and support plans;*
- *The research uncovers gender-specific patterns and the intricate relationship between professional demands and personal life, offering insights crucial for developing tailored interventions that address the complexities of teachers’ well-being*.
